# Beyond victimhood: rethinking communicative resilience and child transformation after parental divorce in Indonesia

**DOI:** 10.3389/fsoc.2025.1668368

**Published:** 2025-11-26

**Authors:** Iis Mardiansyah, Sumardjo, Sarwititi Sarwoprasodjo, Tin Herawati

**Affiliations:** 1 Department of Communication Science and Community Development, IPB University, Bogor, Indonesia; 2 Department of Communication Science, Pamulang University, South Tangerang, Indonesia; 3 Department of Family Science and Consumer, IPB University, Bogor, Indonesia

**Keywords:** communication of resilience, transformative communication, divorce, child identity, meaning negotiation, social stigma, adaptive transformation

## Abstract

This study re-examines the experiences of children of divorced parents within Indonesia’s collectivist and religious context through the lens of the Communication Theory of Resilience and Negotiated Identity Theory. It introduces the Transformative Communication of Resilience Model, which conceptualizes resilience as a communicative, relational, and meaning-making process rather than a static psychological trait. The model identifies five interrelated dimensions–Strategic Construction of Normalcy, Identity Transformation Process, Relational Communication Architecture, Cultural and Religious Meaning-Making, Emotional Negotiation, and the Paradox of Resilience–to explain how children reconstruct identity, reframe trauma, and develop agency through dialog and social interaction. Findings reveal that children are not passive victims but active communicative subjects who negotiate stigma, gendered expectations, and moral judgment while transforming pain into adaptive narratives of growth. Communication functions as both a healing mechanism and a performative act of social resilience, enabling children to redefine their belonging within supportive relational and cultural networks. The practical implications extend to education, counseling, and policy, emphasizing communicative spaces that empower children’s voices and promote emotional literacy, narrative reflection, and cultural inclusivity. Ultimately, resilience is reframed as a transformative communicative act linking trauma to self-redefinition and fostering an inclusive understanding of family and identity in post-divorce contexts.

## 1 Introduction

Divorce has undeniably become a significant social phenomenon in Indonesia’s familial landscape. Recent statistics from the Directorate General of the Religious Courts indicate a notable trend: divorce cases have consistently increased, with more than 400,000 cases documented annually in recent years. Factors contributing to this rise include ongoing disputes, financial hardships, domestic violence, and the prevalence of early-age marriages (Asman and Muda, 2023; Muslih et al., 2023). This increase in divorce rates reflects the evolving dynamics of marital relationships in contemporary Indonesian society, indicating a substantial transformation in the social norms surrounding family life. This conceptual reorientation aligns with recent bibliometric findings that identify social capital and communication as emerging and influential domains in resilience research, underscoring the need for more qualitative and child-centered studies in these areas (Mardiansyah et al., 2024a,b).

The consequences of divorce extend far beyond the couple involved, significantly affecting children, who are often the most vulnerable. Research indicates that children of divorced parents frequently encounter a range of negative emotional, psychological, social, and cognitive impacts. Studies suggest that children may experience feelings of loss, anxiety, rejection, and loyalty conflicts as a result of their parents’ divorce (Ardianto et al., 2024; Bakhita, 2024). Social ostracization can exacerbate these feelings, particularly in a collectivist culture such as Indonesia, where traditional family structures and patriarchal values are esteemed. In this context, divorce is often perceived as a moral failing, contributing to the stigma surrounding divorced families and, by extension, their children (Ardianto et al., 2024). Societal pressures following divorce can complicate children’s adjustment, leading them to face inadvertent questions, accusations, and discriminatory treatment from peers, educators, and community members (Bakhita, 2024). Terms such as *a broken home child* or *divorce victim* can unfairly label these children, impacting their self-esteem and perceptions of societal expectations (Yao, 2023). In conservative communities where familial unity is prized, being identified as a child from a divorced family can restrict social interactions and opportunities, limiting access to community resources, social acceptance, and educational endeavors (Bakhita, 2024).

Given these challenges, communication is a pivotal element in shaping children’s post-divorce resilience and identity. Effective communication allows children to express their emotional experiences, create personal narratives, and redefine their understanding of their family relationships. Interaction through various channels, such as discussions with parents, siblings, friends, or mentors, can facilitate children’s ability to articulate their feelings and seek the emotional support they require during this transitional phase (Portugal and Alberto, 2019). Intrapersonal communication, which involves self-reflection and internal dialog, can also enable children to process their experiences and develop healthier coping mechanisms. Research highlights the importance of viewing resilience as a construct developed through social interactions and narratives (Portugal and Alberto, 2019). This perspective posits that children who recover and thrive post-divorce possess the communicative space necessary to reflect on their experiences, seek support, and reshape their identities through the stories they construct about their lives (Kołodziej-Zaleska et al., 2023).

However, the existing literature often overlooks the role of children as active participants in co-constructing their identities post-divorce. Many studies adopt a pathological view, framing children predominantly as victims in need of aid rather than recognizing their agency in meaning-making and identity negotiation (Asman and Muda, 2023; Kołodziej-Zaleska et al., 2023). This deficit underscores a critical gap in the research, indicating the need for future inquiry that acknowledges children’s capacities to resist stigma and shape their identities through positive social interactions.

Integrating theories such as Resilience Communication and Negotiated Identity enables a deeper understanding of how post-divorce identity formation is an ongoing process shaped by personal and social narratives (Beckmeyer et al., 2022; Watkins et al., 2025). Utilizing these frameworks allows researchers to explore how children navigate their identities amid societal challenges and assert agency in the context of familial dissolution. Moreover, it presents a more holistic view of the post-divorce experience, encouraging a shift from merely surviving parental conflict to actively fostering resilience and adaptive identity construction through communication.

The importance of supportive social environments cannot be overstated; children flourish when surrounded by empathetic adults who foster open dialog about their experiences with them. Programs designed to enhance communication skills within families and communities can strengthen children’s adaptive transformations, guiding them towards a resilient self-perception (Napora, 2019). While divorce in Indonesia may suggest a troubling social trend, it lays the groundwork for critical discussions surrounding children’s experiences and resilience. By recognizing children as active agents in their narratives, future research can lead to interventions that address the emotional fallout of divorce while empowering their voices. Shifting from viewing children solely as victims to acknowledging their roles as communicators and sense-makers is essential for fostering resilient outcomes in post-divorce contexts.

### 1.1 Communication theory of resilience: meaning, narrative, and relationship

Buzzanell (2010) defines communication of resilience as a discursive process through which individuals and groups construct, sustain, and articulate empowerment in the face of a crisis or disruption. Resilience is not understood as an internal, stable capacity possessed by individuals but rather as the outcome of relational and discursive practices that enable people to reconstruct meaning, preserve self-identity, and build supportive social structures.

In this context, Buzzanell identified five fundamental processes involved in the communication of resilience.

1. Crafting normalcy: Children attempt to create a sense of “normal life” despite changes in their family structure. This involves maintaining routines, constructing new family narratives, and preserving a sense of continuity in the family.
2. Affirming identity anchors: Children reinforce meaningful identity points that provide stability and purpose, such as taking on the role of a protective older sibling or excelling as a student.
3. Maintaining and using communication networks—Social support systems, including family members, peers, teachers, and community networks, become crucial communicative resources for navigating emotional distress and sustaining resilience.
4. Putting alternative logic to work: Children develop new and more adaptive narratives about divorce and family life, allowing them to reframe experiences of loss or stigma into stories of growth, learning, and transformation.
5. Foregrounding productive action: Children engage in constructive social behaviors as expressions of agency and empowerment, such as mediating conflicts within the family or volunteering at school.

In the context of children navigating life after divorce, these processes help them rearticulate traumatic experiences into sources of identity development. Crucially, resilience is not a passive reaction but an active meaning-making process shaped by the child’s ability to design and communicate self-narratives that provide new interpretations of their life circumstances.

### 1.2 Negotiated identity theory: identity as a social process

While Buzzanell emphasizes the role of narrative and meaning, Negotiated Identity Theory (Ting-Toomey, 2017) posits that identity is a social process that is continuously negotiated through interpersonal and social interaction. Identity is not fixed; rather, it is shaped and reshaped within complex social relationships, particularly when individuals encounter identity-related tensions or threats.

According to this theory, identity negotiation occurs when individuals do the following:

1. They perceive a mismatch between their personal identity and how they are socially perceived (for example, a child may feel “normal” but is labeled as “broken home” by others).
2. They adapt or assert their identity through either accommodation or reaffirmation strategies.
3. They engage in interactions where their social position (e.g., being a child of divorced parents) is viewed negatively by others.

This negotiation process is not neutral; it unfolds within unequal power dynamics in which dominant norms may suppress or reject the alternative identity narratives that children attempt to construct. As such, a child’s ability to maintain or revise their identity is highly dependent on the availability of safe and supportive dialogical spaces that allow for expression, validation and resistance.

### 1.3 The Indonesian context: collectivism, family norms, and gender roles

In Indonesian society, which is deeply rooted in collectivist values and social harmony, divorce is widely perceived as a deviation from the ideal family norm. The cultural framework of collectivism emphasizes a unified family image as a sacred institution, leading to significant societal judgment when divorce occurs. This judgment results in social stigma not only directed at the separating or divorcing parents but also extending to their children, who may be labeled as “incomplete” or “morally vulnerable,” thereby reinforcing notions of familial failure to conform to established societal expectations (Bakhita, 2024). Research on family dynamics underscores the pervasive impact of cultural norms, revealing how children of divorced parents can be stigmatized as symbols of marital breakdown, which exacerbates their psychological and social struggles in an already challenging situation.

Within this traditional context, children are often subjected to rigid gender roles that further complicate their development and identity. Boys may feel pressured to mature quickly, protect their mothers, and suppress emotional vulnerabilities, whereas girls may be expected to adopt maternal roles or act as mediators in familial conflicts (Dewi et al., 2023). Such gendered expectations can restrict children’s ability to engage in healthy self-expression and identity negotiation. The implications of this social structure result in a narrow pathway for emotional development, where children must grapple not only with divorce itself but also with societal directives concerning masculinity and femininity that dictate how they are supposed to behave during and following such crises.

Communication surfaces as a vital arena for children to resist the stigma associated with their parents’ divorce and construct alternative identities that transcend labels of victimhood. Through active and meaningful communication, children can articulate their experiences and reframe their narratives from *divorced victims* to *survivors* or *agents of change* (Meitasari et al., 2023). The process of identity formation is further enriched when children engage in two-way dialogs with peers, family, and supportive figures, enabling them to process their emotions and reconstruct their understanding of themselves and their family within a broader communal context (Portugal and Alberto, 2019).

The communication process not only affords children the opportunity to reshape their identities but also to navigate the stigmatization they face in their environment. Those who successfully reframe their stories often showcase resilience, not merely as a reaction to their circumstances, but as a proactive stance that emphasizes agency in their lives. Children can confront the narratives imposed upon them by society, actively engaging in the discourse surrounding their identities and experiences, thereby transforming their position within the social fabric.

Importantly, while this communication can serve as a tool for resistance and redefinition, it is often dependent on supportive relationships. Effective communication in families where conflict is prevalent can facilitate healthier family development. Research reveals that children thrive emotionally when they maintain connectivity and open dialog with their custodial parent and have a supportive network that validates their feelings and experiences. This highlights the crucial role of familial and social structures in promoting adaptive strategies for youth navigating the social stigma stemming from divorce.

However, the literature demonstrates a gap in recognizing children as agents of communication and resilience in the context of divorce, particularly in Indonesian culture. While traditional psychological frameworks tend to categorize them as passive victims needing support, a substantial body of evidence suggests that children actively participate in constructing their identities and understanding their situations. They are not merely shaped by their social environments but also possess agency to define their narratives and assert their identities.

Thus, the discourse surrounding children of divorced parents must evolve to embrace a more nuanced understanding of their roles as communicators and resilience-builders. By fostering environments in which their voices are heard and validated, stakeholders can instill a sense of agency in children, allowing them to navigate their post-divorce realities more effectively. This involves acknowledging the challenges posed by societal stigma and recognizing the potential for growth and adaptation that lies within the children themselves.

In conclusion, divorce in Indonesian society presents a complex interplay of cultural norms, social expectations, and individual identities. Caught in this cultural crossfire, children face significant stigma; however, effective communication can provide them with the agency to redefine their experiences. By focusing on their resilience and potential for positive identity construction, society can begin to shift its perceptions, allowing for a more inclusive understanding of family dynamics in the context of divorce. Future research and intervention efforts should prioritize fostering supportive environments that allow children to articulate and reshape their narratives, ultimately leading to more adaptive and empowered identities in the wake of parental separation (Dewi et al., 2023; Meitasari et al., 2023).

### 1.4 Communication as an arena for negotiation and resilience

By integrating Resilience Communication Theory and Negotiated Identity Theory, this study positions children of divorced parents not merely as *wounded subjects*, but also as individuals with the capacity for reflective communication, meaning-making, and identity renegotiation amid social pressure. These two theoretical frameworks offer a more holistic understanding of how children do not simply survive parental divorce but adaptively transform through communicative processes.

Communication, in this sense, is not only a tool for survival but also a performative space, a domain in which children can reconstruct their identities, reclaim meaning, and challenge the stigmas that have constrained them. It is within this communicative arena that resilience takes shape: through narrative reframing, social interaction, and symbolic resistance, children become agents of their own identity formation and social integration.

## 2 Children as agents in post-divorce narratives

Public discourse and much of the academic literature often positions children as passive recipients or victims of divorce. They are frequently portrayed as individuals who must be rescued, healed, or protected, yet are rarely given the space to speak or articulate their own narratives. However, from the perspective of critical communication theory and interpretive approaches, children are not merely the receivers of meaning. Rather, they are communicative agents with the capacity to actively shape, revise, and negotiate meanings.

### 2.1 Repositioning the child: from object to communicative subject

Repositioning children as communicative subjects entails acknowledging their agency in understanding and interpreting the experience of divorce. It is crucial to recognize that children are not merely passive recipients of the divorce process; they actively engage in rearticulating their understanding of the situation from their unique perspectives. This agency manifests in various forms, including narratives, conversation, artistic expression, and social action. Children do not simply internalize parental conflict; rather, they undergo complex cognitive and affective processes to craft a narrative that explains their existence and shapes how they wish to be perceived by those around them (Lestari and Huwae, 2023; Pelleboer-Gunnink et al., 2015).

Moreover, the importance of children’s narratives in the aftermath of divorce cannot be overstated; these narratives encapsulate critical processes of identity negotiation, emotional regulation and meaning reconstruction. Communication is a crucial mechanism through which children can express their feelings and experiences, offering them a path toward understanding their reality while countering the stigma surrounding their family situation (Murniasih and Irvan, 2023; Noor et al., 2023). In environments that may not always be welcoming or understanding of their lived realities, a child’s ability to articulate their thoughts, both literally and symbolically, becomes a vital form of resilience and identity affirmation.

Children actively shape their stories, impacting not only their self-perception but also their emotional well-being in the post-divorce context. By reframing themselves from “divorce victims” to *survivors* or *agents of change*, children illustrate that identity is not merely a static outcome of social structure; rather, it is also a product of reflective communicative agency. Research indicates that social support and healthy communication with caregivers can enhance children’s emotional resilience and help them adapt more successfully to their new circumstances (Murniasih and Irvan, 2023; Russell et al., 2016).

Additionally, exploring the communicative factors at play during these formative experiences reveals the critical role of such supportive relationships. Recent studies have shown that children with healthy, open lines of communication with caregivers and peers are better equipped to navigate the challenges that arise post-divorce (Shabrina et al., 2020; van Spijker et al., 2022). These supportive interactions contribute to strengthening their resilience, providing them with a sense of belonging and understanding that is essential for coping with significant changes in family dynamics.

In this light, communication is not just a tool for sharing experiences; it is an integral aspect of children’s development as they grapple with the implications of divorce itself. The potential for children to express their experiences through various forms of communication, verbal dialog, artistic pursuits, or nonverbal cues can help mitigate feelings of isolation and emotional distress. Recognizing and fostering these pathways can lead to more positive emotional and psychological outcomes during and after the transitions associated with divorce (Bezuidenhout et al., 2018; Yulaf and Semerci, 2019).

As children articulate their experiences, they also become active participants in defining their identities within the context of their families’ restructuring. Through these narrative processes, children assert their agency, allowing them to challenge prevailing narratives surrounding divorce and the stigma associated with it. Research underscores that when children are given the space to express their thoughts and feelings, they develop a more nuanced understanding of their familial situations, which is essential for their psychological resilience and identity formulation (van der Wal et al., 2019; Weaver and Schofield, 2015).

Therefore, understanding child agency in the context of divorce invites a paradigm shift. Rather than viewing children as passive victims requiring protection and intervention, it is essential to consider them as empowered individuals capable of making sense of their experiences and asserting their voices in the narrative of their family dynamics (Weaver and Schofield, 2015). This perspective not only serves to validate children’s experiences but also highlights opportunities for intervention and support that honor their agency and autonomy.

### 2.2 Adaptive communication: narratives, symbols, relationships, and spirituality

Children employ a wide range of adaptive communication forms to navigate the aftermath of divorce. One of the most dominant is the personal narrative, through which children reframe the story of divorce in their own terms, sometimes simplifying it, sometimes avoiding it, and at other times directly challenging the dominant adult-centered narratives. In interviews or journal entries, these narratives may appear in expressions such as: “I do not want to become like them,” “I have to be stronger than this,” or “I want my younger sibling to never feel the same pain.”

Beyond verbal narration, emotional symbols serve as powerful meaning-making tools. For instance, a child may keep a photo of their parents together to preserve the illusion of stability or may discard all memorabilia as a symbolic act of releasing trauma. These symbolic gestures carry emotional weight and communicate inner transformations that may not be easily verbalized by the patient. Social relationships also play a key role in adaptive communication. Interactions with peers, teachers, or surrogate family members often serve as critical relational mechanisms for restoring a child’s sense of identity and belonging. These relationships provide emotional support, alternative role models, and communication contexts for self-expression.

In many cases, children also turn to spiritual communication as a source of strength and meaning in their lives. Prayer, religious reflection, and imagined conversations with God are ways to make sense of suffering, organize hope, and seek existential safety. Here, spirituality functions not only as ritual religiosity but also as an internal communicative resource, a form of transcendent narrative-making that helps children cope with the disruption of family life.

### 2.3 Post-divorce identity forms: survivor, mediator, agent of change

In the process of identity negotiation after parental divorce, children often construct several recurring identity prototypes that reflect their adaptive responses to familial disruption.

#### 2.3.1 The survivor

Children adopting a survivor identity tend to emphasize personal strength and emotional resilience. They express that they have managed to endure the divorce while remaining emotionally intactand, in some cases, have emerged stronger as a result. This identity is rooted in experiences of enduring emotional pain, confronting social stigma, and ultimately rising above it with a narrative such as “This did not break me.

#### 2.3.2 The mediator

Children who assume the role of emotional stabilizers between conflicting parents often feel a strong sense of responsibility to maintain familial harmony. They may mediate disputes, deliver messages between parents, or act as calming influences in the household. Despite the psychological burden, this identity offers children a sense of agency, control, and purpose within the family system, reinforcing their perceived value in emotionally turbulent situations.

#### 2.3.3 The agent of change

In more empowered responses, some children develop the identity of an agent of transformation within the family and in broader social contexts. These children become vocal about the importance of healthy communication, participate actively in community- or school-based initiatives, and often aspire to become counselors, educators, or social advocates. This identity reflects a profound adaptive transformation: they do not merely survive the trauma but reinterpret it as a catalyst for purpose and societal contributions. Their pain is reframed, not as a limitation, but as a source of meaning and social insight.

### 2.4 Reflection and memory as sources of identity narratives

The formation of a post-divorce identity is deeply shaped by personal reflection and social memory. Reflection enables children to revisit past experiences from new perspectives as they grow older, gain emotional maturity, and acquire new information. Both personal and collective memories serve as the foundation for the identity narratives constructed. Children may choose to remember, reinterpret, or deliberately forget certain aspects of the past as part of their strategy for organizing and stabilizing their self-perception.

In this context, time and emotional distance from the divorce event play crucial roles. The identity narrative constructed by a child at age 12 may differ significantly from that constructed at age 25. Thus, post-divorce identity should be understood as open-ended and continuously negotiated rather than as a fixed or final state. It is a dynamic process shaped by evolving reflections, shifting memories and changing social contexts.

## 3 Social and environmental pressures: challenges in identity negotiation

The identity negotiation process for children of divorced parents does not occur in a vacuum or in isolation. As a social construct, identity is continually shaped, challenged, and renegotiated through interactions with one’s surrounding environment. The social environment can function as a supportive force that facilitates the development of a healthy and resilient identity. However, it can also act as an obstructive force, compelling children to conceal, suppress, or rigidly defend their identities in response to stigma and social pressures. In the Indonesian context, deeply rooted in collectivist values, religious norms, and traditional family ideals, the social environment plays a significant role in shaping the terrain on which identity negotiation takes place. Children of divorced parents often navigate a field marked by moral expectations, cultural conformity, and social scrutiny, which can either enable or hinder their capacity to construct and express their evolving sense of self authentically.

### 3.1 The social environment as a current of identity: supportive or restrictive?

The social environment serves as the primary arena in which children experience their existence in a tangible way. From school, peer groups, and neighbors to digital media spaces, these environments become sites where a child’s identity is either affirmed or rejected. A supportive environment can provide children with the space to express themselves, share their stories, and discover meaningful identity anchors. However, when the environment is filled with prejudice and normative judgment, children are often forced to adapt, conceal, or suppress aspects of their identity that are deemed “inappropriate” or “deviant.” In societies that continue to uphold the idealized narrative of the intact, harmonious, religious, and structured nuclear family, children from divorced families are frequently perceived as deviating from this ideal. Their identities are not challenged due to any inherent deficiency but rather because of the social stigma attached to their family’s status. As a result, the child’s identity becomes a site of tension not based on who they are but on how society views where they come from.

### 3.2 Stigma and labeling: from school to social media

Stigma directed at children of divorced parents often manifests in subtle, yet deeply painful, ways. At school, these children may receive differential treatment from their teachers or peers. Some report being mocked or subjected to invasive questions such as, “Why did your parents split up?,” “Who do you live with?,” or “You must be a troublemaker because you are from a broken home.” These narratives inflict symbolic wounds, reinforcing feelings of shame, rejection or inferiority.

Within residential communities, children are often “marked” by their family’s status. Labels such as *“anak janda”* (child of a widow), “anak duda” (child of a widower), or “anak keluarga retak” (child of a broken family) reduce the complexity of their lived experiences to narrow and often pejorative social identifiers. Even in social practices, such as participation in religious events or youth organizations, children from divorced families may encounter implicit exclusion.

Social media further exacerbates these challenges. While digital platforms expand the space for self-representation, they also function as arenas where identity is constructed under the normative social gaze. Children of divorce face the dual pressure of presenting a socially acceptable image in public while concealing aspects of their family background that are deemed inappropriate or unshareable. In some cases, social media becomes a site for the reproduction of stigma through comments, memes, or content that marginalizes or mocks the status of children from non-intact families.

### 3.3 Gender and role expectations in post-divorce dynamics

One of the most significant pressures in children’s identity negotiation after divorce stems from gender-based expectations. In patriarchal cultural contexts, boys are often expected to be “tough,” assume the role of protector for their mothers or younger siblings, and to suppress emotional vulnerability. Expressions of sadness or emotional instability are often perceived as signs of weakness.

In contrast, girls are often prematurely “adultified,” expected to step into the role of the mother, care for siblings, and maintain emotional harmony within the household. These expectations create emotional asymmetry in how boys and girls manage their identities. Boys may suppress emotional expression and construct a façade of strength that is fragile beneath the surface. Girls may forfeit their childhoods, forced into caregiving roles that require them to silence their personal needs in favor of maintaining family stability. Thus, gender is not merely a matter of biological sex but a symbolic structure that shapes how children are positioned within the post-divorce family narrative and, crucially, how they are permitted or constrained in negotiating their identities.

### 3.4 Religion and cultural values: between protection and symbolic oppression

In Indonesia’s deeply religious and culturally rooted society, religion and cultural values often serve as moral references for judging what is “good” or “bad” in social life, including the issue of divorce. On the one hand, religious values can provide children with sources of comfort, meaning, and spirituality. Practices such as prayer, worship, and teachings of compassion frequently function as mechanisms for emotional healing and the development of positive meanings.

Conversely, religion can become a repressive moral framework when used to justify social stigma. Narratives such as “divorce is a sin,” “divorced families are not blessed,” or “children from broken homes must be cautious not to become broken themselves” constitute symbolic oppression. These messages not only blame parents but also pass down a moral burden to the child. Local cultural values can similarly reproduce stigma, especially within communities that strongly uphold customs and family honor. In some cases, children from divorced families are excluded from traditional ceremonies, or their families are seen as having “shamed the ancestors.” At this point, children suffer not only emotional wounds but also cultural exclusion from their peers.

Social pressures emerging from religious, cultural, gendered, educational, and digital environments construct a challenging landscape of identity negotiation for children of divorce. To build resilient and adaptive identities, children require social spaces that are not merely neutral but actively supportive, where they can articulate self-narratives without fear, shame, or threat of stigma. Therefore, interventions for children affected by divorce must go beyond the psychological domain and address communicative and socio-cultural dimensions to deconstruct the symbolic structures that limit their agency.

## 4 Communication of resilience as a pathway to adaptive transformation

In psychological research, resilience has long been understood as an individual’s capacity to withstand or bounce back from stress and adversity. Early approaches tended to view resilience as a personal attribute rooted in inner strength or inherent toughness relatively stable and enduring within the individual. However, contemporary developments in psychological theory have moved well beyond this static view. The fourth wave of resilience studies (Luthar, 2015; Masten et al., 2023; Ungar, 2021) reconceptualizes resilience as a dynamic, ecological, and relational process that emerges through ongoing interactions among biological, psychological, social, and environmental systems. Within this framework, adaptation is no longer understood merely as the outcome of internal traits, but as a probabilistic and contextually embedded developmental process shaped by multiple, interdependent systems.

Although these advancements in psychology have contributed to a more fluid and systemic understanding of resilience, a communication-centered perspectiveas articulated by Buzzanell (2017) extends this logic further by emphasizing the discursive, relational, and meaning-making dimensions of adaptation. From this viewpoint, resilience is not only about surviving disruption, but about reconstructing meaning, identity, and relationships through communication. For children navigating the aftermath of parental divorce, resilience manifests through communicative practices such as storytelling, dialog, and relational engagement that enable them to reinterpret past experiences and author new, contextually grounded identities. Thus, resilience is no longer viewed as a static psychological capacity or merely a multilevel adaptive process, but as a transformative communicative accomplishment a process through which pain and disruption are integrated into narratives of growth, agency, and self-redefinition.

### 4.1 Communication as an arena for narrative and affective reconstruction

Communication serves as the primary medium through which children of divorced parents engage in narrative and affective reconstructions. Through communicative acts, children begin to reframe their understanding of who they are, what family means to them, and how they interpret their past and imagined future. This process is rarely linear or conflict-free in nature. Children may experience ambivalence, sadness, or even anger when attempting to rearticulate their life stories. However, within this struggle lies a profound opportunity to rebuild a more resilient and meaningful sense of self.

Personal narratives play a central role in this study. When children are given the space to tell their stories in full, including the painful, confusing, and contradictory parts, they begin to reorganize their emotional responses and internal logic. This process, often referred to as re-authoring, involves rewriting one’s life story to extract new meaning and reconfigure the emotional structure.

Here, communication becomes not only a cognitive tool but also an affective process that touches the deepest layers of emotion and existential selfhood. It allows children to engage in healing not just through telling but through feeling, reframing, and re-owning their narratives with agency and voice.

### 4.2 Communication patterns and safe spaces for identity negotiation

Adaptive identity transformation cannot occur without the presence of safe social environments where children feel accepted, heard, and free from judgment. These spaces may emerge through communication with supportive parents, empathetic teachers, understanding peers, and inclusive online communities.

Communication patterns are crucial within these spaces. Participatory, validating, and reflective forms of communication facilitate emotional recovery and identity negotiations. In contrast, judgmental, dismissive, or stigmatizing communication reinforces psychological wounds and closes off opportunities for growth, whereas safe spaces enable children to explore multiple identity possibilities, not simply to choose one, but to negotiate, integrate, or even invent hybrid identities that better reflect their lived experiences and aspirations. It is within these supportive environments that children can assert: “I am not just a child of divorce; I am also a survivor, a mediator, and an agent of change.”

### 4.3 Proposed model: transformative communication of resilience model

Based on the conceptual findings and theoretical reflections presented in the previous sections, this article proposes the Transformative Communication of Resilience Model, which explains how children of divorced parents undergo adaptive transformation through communicative negotiation of meaning and identity.

**Figure 1:**
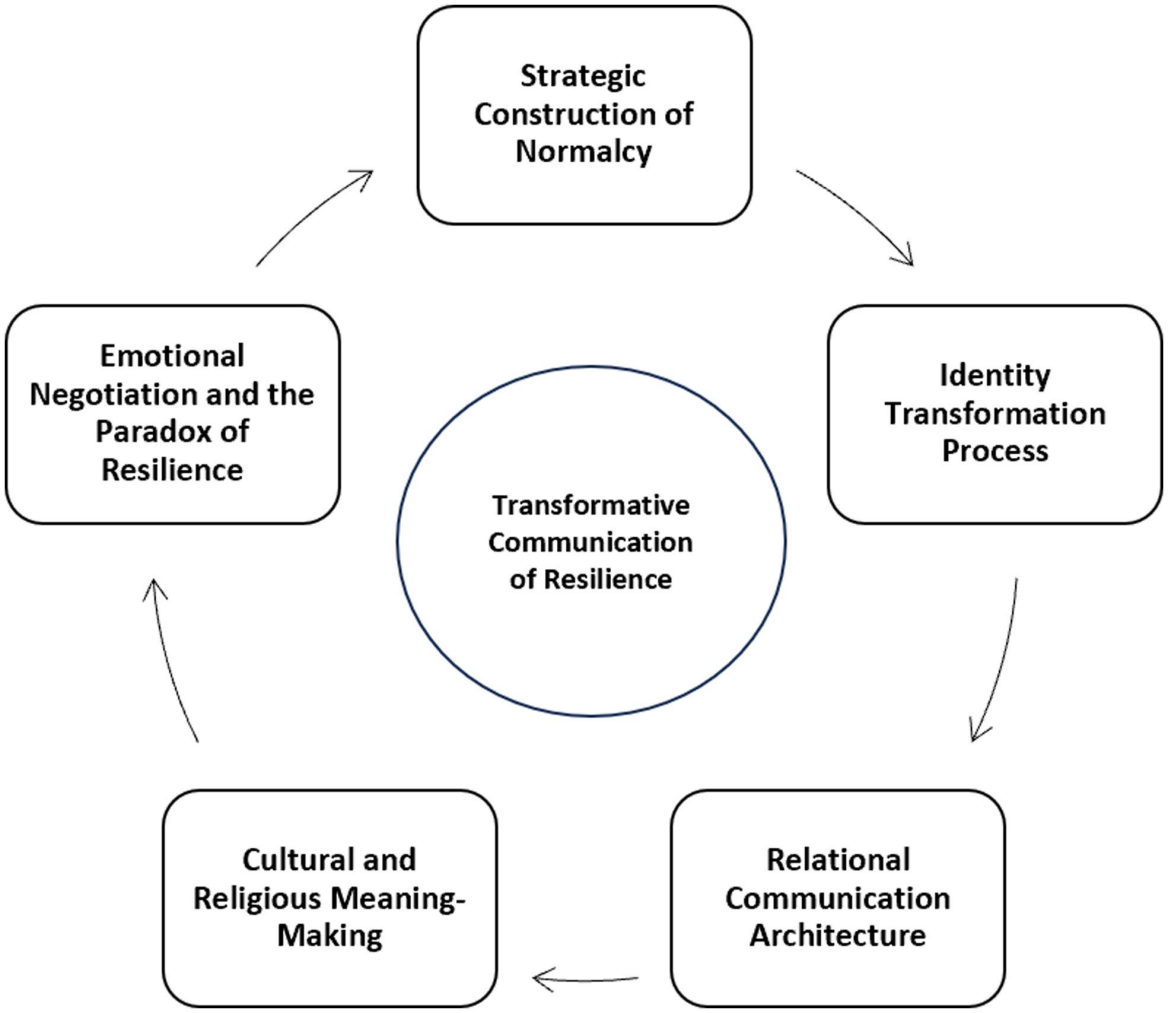
Transformative communication of resilience model.

Figure 1 illustrates the Transformative Communication of Resilience Model, which conceptualizes resilience among children experiencing parental divorce as a dynamic, relational, and cyclical process. At the center of the model lies the Transformative Communication of Resilience, representing the communicative arena in which children reinterpret family disruption, negotiate their emotions, and reconstruct new meanings of self and belonging. Communication here is not merely a medium of coping, but the core mechanism of transformation through which trauma is translated into growth, vulnerability into agency, and disconnection into reconnection.

Surrounding this central communicative process are five interrelated dimensions that describe the iterative pathways of post-divorce identity formation and meaning-making:

1. Strategic Construction of Normalcy – children’s efforts to recreate a sense of order, stability, and “normal life” after family disruption, redefining what normalcy means within their new familial structure.
2. Identity Transformation Process – the ongoing process of self-redefinition, where children reframe their experiences and shift from seeing themselves as victims to viewing themselves as survivors, mediators, or agents of change.
3. Relational Communication Architecture – The social infrastructure of communication, including parents, peers, teachers, and community members, that provides emotional validation and supports resilience through dialog and empathy.
4. Cultural and Religious Meaning-Making – the interpretive process through which children draw upon spiritual, cultural, and moral frameworks to construct meaning from adversity, integrating their experiences within broader social narratives.
5. Emotional Negotiation and the Paradox of Resilience: The emotional dimension of resilience, where children navigate ambivalence, pain, and hope, acknowledging that resilience involves both struggle and transformation, rather than linear recovery.

Together, these five dimensions form a circular and interactive system that emphasizes the continuous interplay between emotional, relational, and cultural forces. This model highlights that children’s post-divorce resilience and identity are not formed instantly or linearly but emerge through ongoing communicative engagement that connects past wounds with future possibilities. In this sense, communication serves as a bridge between trauma and transformation, enabling children to reconstruct meaning, renegotiate self-concepts, and cultivate adaptive, empowered identities within their evolving social and cultural environments.

### 4.4 Communication as an act of social resilience

Within the framework of the Transformative Communication of Resilience Model, communication extends beyond individual coping mechanisms to function as a collective and transformative social act. When children of divorced parents articulate their experiences through dialog, storytelling, and symbolic expression, they not only reconstruct personal meaning but also participate in reshaping societal narratives surrounding divorce. Through communicative engagement, children transform silence into voice and stigma into agency, demonstrating that resilience is both relational and performative. Their narratives become acts of symbolic resistance that challenge the cultural norms, gender expectations, and moral judgments embedded in Indonesian society. In this sense, the communicative process fosters social resilience, generating empathy, inclusivity, and broader cultural awareness. Communication thus operates as a transformative bridge between personal recovery and social change, where children’s voices inspire communities to reimagine family, identity, and belonging beyond the boundaries of stigma.

## 5 Conclusion and perspective implications

Parental divorce disrupts not only the family structure but also the meanings that shape a child’s identity and emotional world. Grounded in the Transformative Communication of Resilience Model, this study emphasizes that children are not passive victims but active communicative agents who reconstruct meaning, negotiate identity, and transform pain into growth through dialog, reflection, and relational engagement. Communication emerges as the central arena where resilience is cultivated, linking trauma to transformation and enabling children to redefine selfhood and belonging. Their identities evolve through ongoing interactions with social, emotional, and cultural forces, manifesting as survivors, mediators, or agents of change. These roles illustrate that resilience is not static but a dynamic, dialogic journey shaped by relational communication and social validation. Ultimately, communicative resilience transcends psychological recovery, functioning as a transformative process that empowers children to rebuild meaning and contribute to a more inclusive understanding of family and identity.

### 5.1 Practical implications

The practical recommendations derived from the Transformative Communication of Resilience Model are grounded in its five interrelated communicative dimensions Strategic Construction of Normalcy, Identity Transformation Process, Relational Communication Architecture, Cultural and Religious Meaning-Making, Emotional Negotiation, and the Paradox of Resilience. Each dimension informs specific strategies across education, counseling, and policy to foster environments that support children’s communicative, emotional, and social transformations after parental divorce. These recommendations, therefore, extend beyond psychological recovery toward cultivating reflective, relational, and culturally responsive practices that align with the model’s emphasis on communication as the bridge between trauma and transformation.

#### 5.1.1 Implications for education

In educational settings, the model underscores the importance of reflective narrative communication as part of children’s meaning-making process. Teachers and school counselors should create safe dialogic spaces where children can express their experiences through storytelling, art, or discussion, allowing them to reconstruct meaning and normalize their family situation (*Strategic Construction of Normalcy*). Integrating emotional literacy programs and classroom-based narrative reflection encourages self-awareness and identity growth consistent with the model’s *Identity Transformation Process.* By recognizing communication as a form of resilience, schools can become environments where students are not labeled by their family background but are empowered to reinterpret it.

#### 5.1.2 Implications for family counseling and psychosocial practice

The Transformative Communication of Resilience Model positions counseling as a dialogic and participatory space for identity re-authoring. Counselors should focus on narrative reconstruction and emotional negotiation, guiding children to externalize painful experiences and reframe them into stories of strength and renewal (Emotional Negotiation and the Paradox of Resilience). Techniques such as reflective journaling, family dialog sessions, and creative expression align with the model’s relational and communicative foundation (Relational Communication Architecture). Moreover, integrating cultural and spiritual frameworks into counseling (Cultural and Religious Meaning-Making) can help children draw from familiar moral and faith-based narratives to rebuild coherence and hope within their lived context.

#### 5.1.3 Implications for policy and institutional design

At the policy level, the model emphasizes the creation of relationally supportive environments that sustain children’s communicative resilience across multiple systems such as family, school, community, and digital spaces. Policymakers should design family centered and child agency-oriented programs that promote open dialog and narrative-based practices within family support and education initiatives. Policies should explicitly recognize communication as a developmental resource, ensuring that children from divorced families have equitable access to psychosocial services, peer support forums, and culturally sensitive interventions. This approach reflects the model’s Relational Communication Architecture and Cultural and Religious Meaning-Making dimensions, translating theory into a systemic action that bridges social, educational, and spiritual support structures.

Collectively, these recommendations operationalize the Transformative Communication of Resilience Model by transforming theory into an actionable practice. Education promotes reflection and normalization, counseling facilitates emotional and narrative reconstruction, and policy fosters relational and cultural connectivity. Together, they embody the model’s communicative principle that resilience is not the restoration of the past but the transformation of meaning through ongoing interaction, empathy, and dialog. This approach enables children of divorced parents to move from silence to self-expression, stigma to self-worth, and disruption to empowered transformation.

### 5.2 Recommendations for future research

Future research should expand the understanding of children’s post-divorce resilience by integrating the communicative, cultural, and structural dimensions that shape identity negotiation. Moving beyond psychological and social frameworks, scholars are encouraged to adopt transdisciplinary approaches, such as narrative inquiry, ethnography of communication, and phenomenology, to capture the emotional and relational complexities of children’s lived experiences. Comparative studies should explore how urban–rural differences, socioeconomic status, gender norms, and religious cultural values influence the formation of resilient identities. In Indonesia, where divorce remains stigmatized, investigating how children construct meaning and agency through dialog and symbolic communication will provide valuable insights into their adaptive processes. Future research must position children as active communicative agents rather than passive subjects, emphasizing communication as a transformative space in which identity, resilience, and belonging are continually renegotiated within evolving family and social contexts.
